# Biotechnological lactic acid production from low-cost renewable sources *via* anaerobic microbial processes

**DOI:** 10.5114/bta.2024.139757

**Published:** 2024-06-25

**Authors:** Andriy Anta Kacaribu, Darwin

**Affiliations:** 1 Department of Agricultural Industrial Technology, Universitas Syiah Kuala, Banda Aceh, Indonesia; 2 Department of Agricultural Engineering, Universitas Syiah Kuala, Banda Aceh, Indonesia

**Keywords:** lactic acid, microbial fermentation, low-cost renewable sources, substrate and product inhibition, purification techniques

## Abstract

Lactic acid (LA) production from microbial fermentation using low-cost renewable sources has emerged as an attractive alternative to the use of petroleum-based products. This approach not only offers sustainable solutions for waste management but also enables the production of value-added products in an eco-friendly manner. However, to make this approach economically viable, optimizing the production process for high yield, productivity, and purity while minimizing costs is crucial. To address these challenges, various approaches have been proposed, including the use of neutralizing agents, high cell density cultures, co-cultures, fed-batch fermentation, and product removal strategies. Overall, this review underscores the potential of microbial fermentation for LA production as a sustainable and cost-effective solution to meet the growing demand for eco-friendly products. Further optimization of fermentation processes and the development of new microbial strains and fermentation techniques are key to advancing this approach. The production of LA through microbial fermentation presents a sustainable and eco-friendly solution to the increasing demand for eco-friendly products. With continued innovation, we can expect to see a significant reduction in the environmental impact of industrial processes, coupled with a more cost-effective and high-purity source of lactic acid for various industries.

## Introduction

Currently, there is a significant focus on utilizing renewable natural resources as raw materials for fermentation. This approach is favored for its ability to reduce environmental pollution, its sustainability, and its cost. The raw material sources can be obtained from agricultural products or waste that isrich in carbohydrates (Darwin et al., 2019a). The fermentation process is assisted by microorganisms that have been cultured or uncultured microorganisms. This process can produce various bioproducts such as lactic acid (LA), ethanol, methane, hydrogen, volatile fatty acids, and amino acids (Darwin et al., 2021).LA production through fermentation is being intensively carried out because of its great benefits. LA finds use in the food industry as a preservative, buffer solution, and acidulant(John et al., 2007; John et al., 2009; Satyanarayana et al., 2012; Wang et al., 2015; Ahmad et al. 2020; Mora-Villalobos et al., 2020), among other uses.Additionally, LA is utilized in the textile, pharmaceutical, packaging (polylactic acid), and chemical industries, where it serves as a precursor for various chemicals like propylene glycol, propionic acid, acrylic acid, and acetaldehyde) (Cui et al., 2011; Wang et al., 2015; Li et al., 2021).

**Fig. 1:**
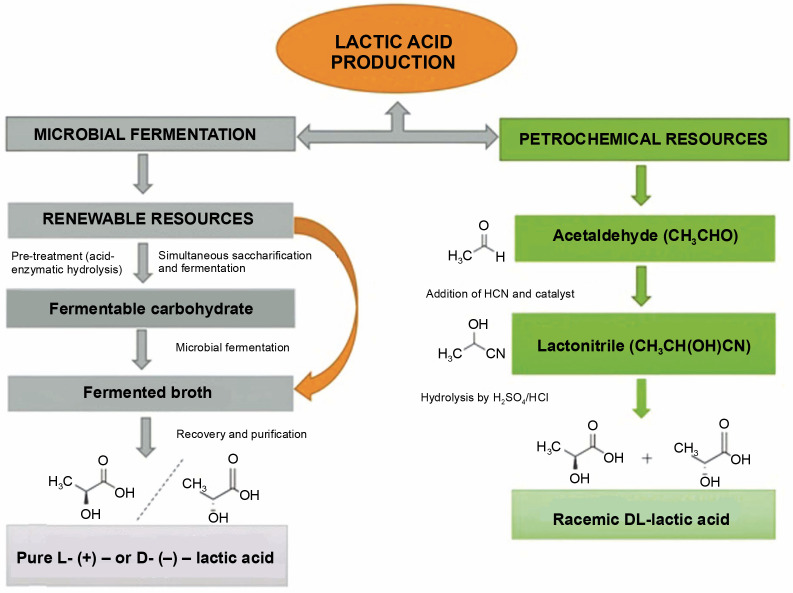
Scheme of lactic acid production *via* microbial and chemical synthesis (Wee et al., 2006)

There are two ways to produce LA, chemical synthesis and microbial fermentation, as shown in Figure 1. The chemical synthesis method generates environmental pollution and high cost. Petrochemicals are employed as raw materials in this process. However,the method yields impure LA (racemic L and D LA) (Castillo Martinez et al., 2013). In contrast, microbial fermentation offers a more eco-friendly and cost-effective approach, producing pure LA (D/L-LA). Currently, around 90% of LA is produced using microbial fermentation due to its accessibility and affordability (Wee et al., 2006; Abdel-Rahman et al., 2011; Boontawan et al., 2011; Alves de Oliveira et al., 2018; Tarraran and Mazzoli, 2018). Despite its advantages, microbial fermentation for LA production faces challenges such as high production costs, low yield and productivity, and difficulties in purifying and recovering LA from the fermentation broth. Addressing these challenges is crucial for maximizing the potential of microbial fermentation in LA production.

## Lactic acid fermentation feedstock

Fermentation using renewable substrates can be utilized due to its low cost, reduced economic losses, abundant, high sugar content, and because it is not competitive with the food sector.

### Lignocellulosic biomass

Lignocellulosic biomass is a natural material that can be used as a raw material for LA production. Lignocellulosic biomass can be obtained from sources like agricultural and industrial waste such as bagasse, grass, straw, corn cob, wood waste, molasses, sugar beet pulp, and coconut pulp (Ghaffar et al., 2014; Darwin et al., 2018; Alexandri et al., 2022). However, utilizing lignocellulosic biomass for LA production typically requires pretreatment to break down the complex structure of lignocellulose into fermentable sugars like glucose and sucrose. Pretreatment methods include physical, biochemical, thermodynamic, and catalytic processes. However, without pretreatment, lignocellulosic fermentation can still produce LA, but with a lower yield (Abdel-Rahman et al., 2011; Velvizhi et al., 2022).

### Food waste

Food waste is a significant issue, with about 95% of food scraps being discarded and ending up in waste disposal sites. However, food waste can be a valuable resource if handled properly (Uçkun et al., 2014; Wen et al., 2016; Breunig et al., 2017). It contains a significant amount of carbohydrates, (Tang et al. 2016; Darwin, 2019; López-Gómez et al. 2020)including sugars and starch, which can be utilized as a substrate for the production of various bio-products, including lactic acid. Sources of food waste include households, cafeterias, restaurants, bakeries, and food industries (Kim et al., 2003).

### Sugar and starch materials

Molasses is a by-product of sugar production from sugarcane. Molasses contains high levels (about 40–60%) of simple sugars (glucose, sucrose, and fructose), inorganic compounds, vitamins, and proteins. However, it also contains inhibitors such as phenolic compounds and metal ions that can interfere with the fermentation process. Pretreatment methods are needed to remove these inhibitors and improve the efficiency of fermentation (Vignesh et al., 2022). In addition to molasses, whey can also be used as a fermentation substrate because it contains high-energy proteins and milk sugars (lactose) (Alonso et al., 2010). In addition to molasses and whey, agricultural fruit waste, including unmarketable pear fruit including unripe, damaged, and over-ripe, tomato pomace seeds and skins (Costa et al., 2021), and coffee pulp (Pleissner et al., 2016).

Starch is an organic material that is rich in sugar content, but direct fermentation of starch produces LA products with low percentage yield and other products such as alcohol. Thus pretreatment (hydrolysis) of starch into simple sugars with the help of saccharifying enzymes such as α/β-amylase and glucoamylase is needed to obtain high-yield LA (Chu-Ky et al., 2016; Darwin, 2019). Many studies have reported the use of starch waste as a fermentation substrate such as Cassava and rice (Odey et al., 2018), a mixture of cassavarice bran and soluble starch (Sharma et al., 2020), *T*. *durum* wheat (Alfonzo et al., 2017), and potato skin waste (Liang et al., 2015).

### Macroalgae and microalgae

Macroalgae, also known as seaweed, are multicellular photosynthetic organisms commonly found in the ocean. They offer several advantages as a raw material for lactic acid production, including a high carbohydrate content, no need for fertile land or freshwater, richness in nutrients, low lignin content (3–7.3% compared to the 25–35% in lignocellulosic biomass), and a fast growth rate (Cesário et al., 2018; Chung et al., 2021; Filote et al., 2021). Microalgae, on the other hand, have also garnered attention as a raw material for lactic acid production due to their ability to accumulate carbohydrates in their cells up to 50–70% (dry weight basis) and their cell walls consisting of hemicellulose and cellulose with no lignin content. Microalgae are also rich in other compounds such as pigments, proteins, carotenoids, lipids, vitamins, and steroids (Chen et al., 2013; Garofalo et al., 2022). Before the fermentation process, both substrates require pretreatment with thermal acid hydrolysis to produce fermentable sugars (Talukder et al., 2012; Tong et al., 2021).

### Glycerol

Glycerol is a by-product of the biodiesel, bioethanol, and oleochemical industries. Biodiesel production typically yields approximately 1 kg of glycerol for every 10 kg of biodiesel, while bioethanol production results in a 10% by-weight glycerol by-product. Many researchers have shown that lactic acid bacteria can produce lactic acid from glycerol (Murakami et al., 2016; Doi, 2018). Apart from using microorganisms (LAB) in the production of LAfrom glycerol, researchers have also developed other methods to convert glycerol into LAvia chemical reactions (oxidation reactions) in the presence of catalysts and enzymes (Tao et al., 2016; Ma et al., 2022).

## Microorganismsfor lactic acid production

There are many types of microorganisms capable of producing LA from various substrates through fermentation, including bacteria, yeast, algae, and fungi. Among these, bacteria are the most commonly used, with lactic acid bacteria (LAB) being the most frequently employed genera (Zheng et al., 2020; Wang et al., 2021). LAB belongs to the *Lactobacillales* which consist of *Lactobacillus*, *Aerococcus*, *Pediococcus*, *Enterococcus*, *Carnobacterium*, *Vagococcus*, *Tetragenococcus*, *Oenococcus*, *Leuconostoc*, *Streptococcus*, *Weissela*, and *Lactococcus* (Hofvendahl and Hahn-Hägerdal, 2000; Sedó et al., 2022).

**Fig. 2:**
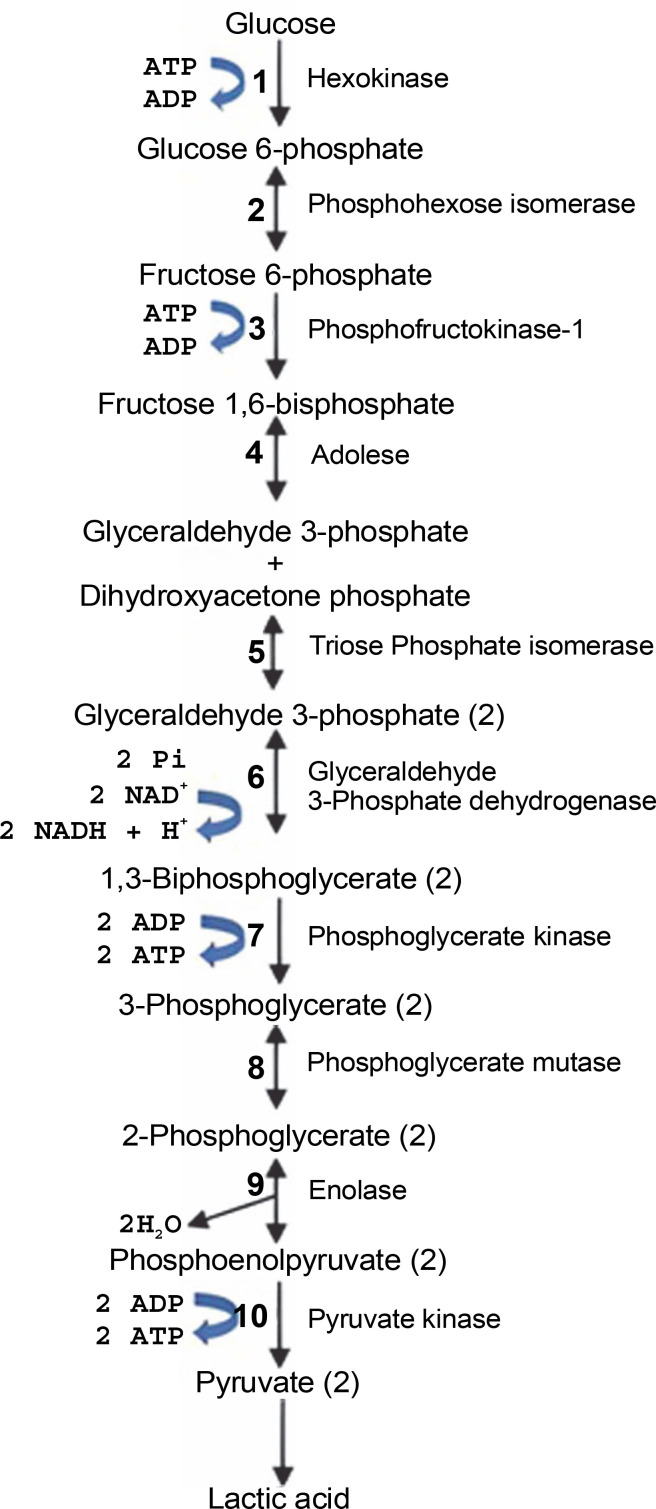
Lactic acid production *via* EMP pathway


LABsare categorized into two types: homofermentative and heterofermentative strains. Fermentation with homofermentative LAB proceeds *via* the Embden-Meyerhof-Parnas pathway (EMP pathway), as shown in Figure 2, where LA is the main product. During fermentation, LAB converts 1 mol of glucose to pyruvate *via* glycolysis and then produces 2 mol of LA and 2 mol of energy (ATP) according to equation (1) (Castillo Martinez et al., 2013; Eiteman and Ramalingam, 2015; Grewal et al., 2020). Homofermentative LAB include *Lactococcus*, *Pediococcus*, *Streptococcus*, *Enterococcus*, and some types of *Lactobacillus* (Andersen et al., 2001).

$${\text{C}}_{6}{\text{H}}_{12}{\text{O}}_{6}\to 2\text{LA}+2\text{ATP}$$ (1)

**Fig. 3:**
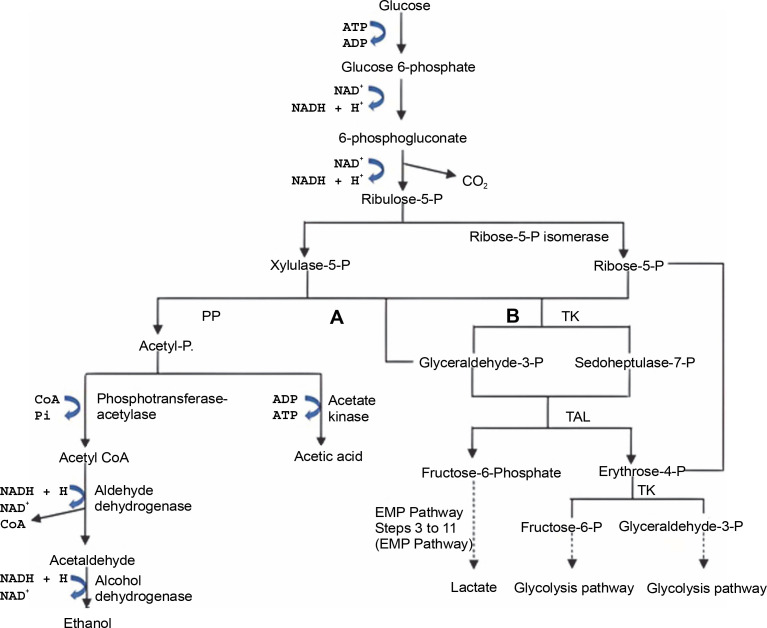
Lactic acid production *via* PK (A) and PP (B) pathway

Not all homofermentative LAB convert glucose to LA *via* the EMP pathway; some use the pentose phosphate (PP) pathway, as shown in Figure 3. In this process, pentoses, and hexoses produce 1.67 mol of LA and 1.67 mol of ATP from hexoses or 0.67 mol of ATP from pentoses (Cubas-Cano et al., 2018). Unlike homofermentative LAB, heterofermentative LAB proceeds through the phosphoketolase(PK) pathway, converting glucose into 1 mol of LA, ethanol, and CO_2_,as shown in Figure 3. This type of LAB belongs to the family Leuconostocaceae and some species of the genus *Lactobacillus* (Endo and Dicks, 2014; Eiteman and Ramalingam, 2015; Borreani et al., 2018).

In addition to cultured bacteria, LA can also be produced using undefined mixed microbial cultures such as rumen, activated sludge, and anaerobic sludge (Darwin et al.,2019b). LA fermentation under nonsterile conditions with undefined mixed microbial cultures by batch and continuous mode fermentation with the addition of nutrients has been reported (Tang et al., 2017). Microorganisms commonly found in the rumen include archaea, bacteria, protozoa, fungi, and viruses (Wang et al., 2018; Lobo and Faciola, 2021). The types of bacteria present in the rumen affect the fermentation products of carbohydrates. Bacteria that play a role in rumen fermentation include *Streptococcus bovis*, *Selenomonas ruminantium*, and *Prevotella bryantii*. Studies have shown that rumen bacteria not only produce lactic acid but also volatile fatty acids and ethanol as metabolites (Belanche et al., 2012). Similar to the rumen, activated and anaerobic sludge also contains many microorganisms that can be used as unsterile cultures in the fermentation process (Darwin, 2019). The use of undefined mixed microbial cultures as unsterilized cultures offers promising prospects for producing LA. However, determining appropriate operating parameters is still necessary to accumulate LA in large quantities. In addition, developing a purification mechanism for the obtained LA is also necessary.

## Methods used to produce LA

Many fermentation processes for producing LA from renewable substrates using different approaches have been reported by researchers, as summarized in Table 1. The effectiveness of LA fermentation can be altered by changing the design of the operating mode used during fermentation, including batch, fed-batch, and continuous modes. Each operating mode has its advantages and disadvantages. Batch mode fermentation typically generates a high yield of lactic acid while the continuous system may enhanceits productivity (Pleissner et al., 2017). Further, batch fermentation is the most widely used method because it is easy to operate, has less contaminant, and with increased titer. However, this mode has drawbacks such as low productivity and inhibition due to substrate and end-product accumulation (Rawoof et al., 2021). Fed-batch fermentation is also widely used because of fewerissues resulting from substrate inhibition and increased titer. This mode has its disadvantages such as inhibition due to the end product and difficulty in maintaining the optimal parameters of fermentation (Huang et al., 2023). Besides, continuous operation is also frequently employed. This is because it can yield high productivity of the fermentation end-product, and also could control the growth of biomass (Ahring et al., 2016). The determination of the operational mode is based on the substrate price and capital available in LA production (John et al., 2007). Choosing the appropriate operational mode can help improve LA production productivity.

**Table 1:** Summary of LA production from different fermentation approaches and processes with renewable feedstock

Microorganism	Substrates	Fermentation mode	Lactic acid			References
			C [g/l]	Y [g/g]	P [g/l/h]	
Marine-animal-resources (MAR)	kitchen refuse	batch	39.2	–	1.09	(Tashiro et al., 2016)
*L. casei* Shirota	mixed food waste	batch	94	0.94	2.61	(Kwan et al., 2016)
	bakery waste		82.6	0.94	2.50	
*B. coagulans*	coffee mucilage	batch	> 40	0.70–0.77	4–5	(Neu et al., 2016)
*B. coagulants*	coffee pulp	batch	83.43 ± 3.66	0.54 ± 0.04	3.57 ± 0.87	(Pleissner et al., 2016)
*Bacillus coagulans*	bakery waste	batch	62.2	0.57	2.59	(Alexandri et al., 2020)
	bakery waste and lucerne	batch	79.09	2.66	0.69	
*L*. p*lantarum* 23	glucose	batch	34.19	0.87	4.57	(Chen et al., 2020)
		continuous (HRT= 4 h)	31.75	0.93	7.94	
		continuous (HRT = 2 h)	28.45	0.94	14.22	
	microalgae	batch	42.34	0.93	7.56	
		continuous (HRT = 4 h)	39.72	0.99	9.93	
L. *delbrueckii* ssp. *delbrueckii* CECT 286	orange peel waste	batch	45	86	–	(Bustamante et al., 2020)
L. *delbrueckii* ssp. *bulgaris* CECT 5037			39	84	–	
*B. coagulans*and *L. rhamnosus*	cassava bagasse	batch	112.5	0.88	2.74	(Chen et al., 2020)
*Kosakoniacowanii* (24 h)	agro-industrial waste	batch	24.97	–	–	(El-Sheshtawy et al., 2022)
*Kosakoniacowanii* (48 h)			27.91	–	–	
*L. helveticus*	cheese whey	batch	53.0	–	–	(Soriano-Perez et al., 2012)
*Entrococcus faecium* FW26	food waste and banana peels	batch	33.3	0.84	0.28	(Abdel-Rahman, S.E.D. Hassan et
*Entrococcusdurans* BP130	food wastes	batch	28.8	0.85	0.24	(Hassan et al., 2019)
*Rhizopus oryzae*	sophoraflavescens residues	batch (SSF)	46.78	–	0.97	(Ma et al., 2020)
*Streptococcus sp*.	food waste	batch	50.0	–	2.93	(Peinemann et al., 2019)
		continuous	69.0	–	1.27	
*B. coagulans* 36D1	cellulose	fed-batch (SSF)	80.0	0.8	0.3	(Ou et al., 2011)
*B. coagulans* strain H-1	corncob residue	fed-batch (SSF)	79.10	0.76	–	(Jiang et al., 2019)
		batch (SFF)	68.0	0.85	–	
*L. rhamnosus*	carob waste	batch	40.0	66.6	1.66	(Bahry et al., 2019)
*B. coagulans*	sweet sorgum juice	batch	78.75	0.78	1.77	(Olszewska-Widdrat et al., 2019)
		batch (PS)	73.0	0.70	1.47	
*L. delbrueckii* NCIMB	beet molasses	batch	90.0	0.97	3.8	(Kotzamanidis et al., 2002)
*L. delbrueckii*	Alfalfa fibers	batch	35.4	0.35	0.75	(Sreenath et al., 2001)
*Rhizopus microsporus*	cassava starch	batch (SSF)	-	0.84	–	(Trakarnpaiboon et al., 2017)
		fed-batch (SSF)	105–119	0.93	1.25	
*L. casei*	sugarcane molasses	batch	120.23	0.91	–	(Thakur et al., 2019)
*L. caseishirota*	food waste	batch	94.0	0.90	2.5	(Kwan et al., 2017)
	confectionery waste		82.6	0.94	2.5	
*E. coli*	glycerol	fed-batch	111.0	0.78	2.8	(Kangming Tian, 2012)
Indigenous microorganism	biowaste	batch	40.6	1.04	1.69	(Probst et al., 2015)
*Halolactibacillushalophilus*	sucrose	batch	65.0	0.83	1.1	(Calabia et al., 2011)
*L. paracasei*	algae	batch (SSF)	37	0.46	1.03	(Nguyen et al., 2012)
*L. pentosus*	corn stover	fed-batch	74.8	0.65	0.7	(Zhu et al., 2007)
*L. rhamnosus*	lactose from whey	fed-batch	106.2	–	1.77	(Bernardo et al., 2016)
		batch	57.0	–	1.18	
*L*. sp. G-02 and *A. niger* SL-09	artichoke flour	co-culture (SSF)	120.5	0.95	3.35	(Ge et al., 2009)
*S. laevolacticus*	raw sugar cane	batch	77.0	0.88	0.86	(Sawai et al., 2011)
		cont. culture	67	0.96	12.20	
*Rhizopus oryzae*	potato starch	batch with NA-CaCO_3_	43.3	–	1.23	(Yen et al., 2010)
		batch with NA-NaOH	41.2	–	0.48	
		batch with NA-NaHCO_3_	35.5	–	1.14	
		batch with NA-ammoniacal	25	–	0.82	

C – concentration, Y – yield, P – productivity, HRT – hydraulic retention time, h – hours, SSF – solid-state fermentation, PS – pilot scale, NA – neutralizing agents

## Factors affecting lactic acid production

### Temperature

Temperature is a critical factor to consider in LA production, as it affects the activity of cell enzymes and the overall metabolism of LA production. Enzymes can only be fully active at their optimal temperature, ensuring that enzymatic reactions occur at maximum rates. Deviations from the optimal temperature can lead to changes in cell metabolism (Panesar et al., 2010). Fermentation can be conducted at various temperatures, including ambient, mesophilic, thermophilic, extreme thermophilic, and hyperthermophilic temperatures (Peinemann and Pleissner, 2020). Each bacterial strain has a different optimal operating temperature for growth (Kim et al., 2012). Bacterial species have specific characteristics, and they cannot grow at temperatures that are not suitable for their characteristics. Therefore, it is crucial to consider the proper optimal temperature during the fermentation process (Tango and Ghaly, 1999). In the fermentation process, it is common practice to usean inoculum and substrate capable of with standing elevated temperatures. These components can operate at temperatures higher than those typical in standard microbial fermentation and exhibit a strong capacity for lactic acid production. However, using temperatures above room temperature requires external energy input, which increasesthecosts of the fermentation process.

### pH

pH is a crucial factor to consider during the LA fermentation process. The optimal pH range for fermentation typically ranges from 4 to 6. pH can be controlled at the beginning of the process and allowed to change as LA is formed (Darwin et al.,2019b). Control of pH can be achieved by adding neutralizing agents or by separating the formed LA from the fermentation broth using appropriate separation methods such as electrodialysis, extraction, or adsorption (Hofvendahl and Hahn-Hägerdal, 2000; Hetényi et al., 2011). Proper pH adjustment is necessary as it can affect bacterial growth rate and LA production productivity. Various types of agents (bases) can be added to adjust the optimal pH (Peeva and Peev, 1997). Several researchershave reported theuse of various neutralizing agents such as KOH, NaOH, NH_4_OH, CaCO_3_, (CH_3_)_2_NH, and N(CH_3_)_3_ (Vasiljevic et al., 2005; Akalɪn et al., 2017; Hajilary et al., 2019). LA fermentation can be carried out without pH control, but the resulting productivity mayvary, with lower production costs but still facing many challenges (Abdel-Rahman and Sonomoto, 2016; Pau et al., 2022).

### Nutrients

Many researchers have explained, based on phenotypic characteristics and genomic analysis, that each species of microorganism cannot grow with just a carbon source without appropriate mineral nutrient supplements in the media. Hence, the media should contain nutrients such as whey proteins, amino acids as a nitrogen source, oligosaccharides, lipids,minerals, and buffering agents to support the growth of microbes (Blaiotta et al., 2017; Hayek et al., 2019). Nutrients are essential in LA fermentation, as they facilitate the growth of bacteria and increase the productivity of LA production.

Fermentation carried out with lowamounts of nutrients and high substrate levels can lead to obstacles in producing LA. Therefore, an appropriate composition ratio of the abovementioned components is needed for the fermentation to proceed well. Various types of nutrients are commonly used by researchers, such as rogosa agar, MRS medium, skim milk, whey agar, and M17 medium (Blaiotta et al., 2017; Veselá et al., 2019). However, the production of such media requires expensive ingredients. Thus, the development of low-cost media from easily obtainable materials is needed to reduce the cost of LA production. Another alternative is the development of lactic acid bacteria strains that require small amounts of nutrients and can convert carbohydrates into LA (Kadam et al., 2006).

### Inoculum size

In studies of LA fermentation, the size of the inoculums (the population of microbes introduced into the fermentation medium) typically ranges from a percentage of the total volume of the fermentation broth (i.e., 5–20%). A larger inoculum size used during the fermentation process results in faster and larger concentrations of LA produced (Darwin et al., 2019b). This is because a higher inoculum size leads to a lower initial pH (acidic), which facilitates easier and quicker lactic acid accumulation, resulting in high yield and productivity (Panesar et al., 2010; Taleghani et al., 2016; Wardani et al., 2017).

For example, the production of LA from *L. casei* with inoculum sizes ranging from 5 to 20% yields different results. Using a 20% inoculum (1-day-old inoculum) resulted in a higher lactic acid yield (93%) with a productivity of 8.8 g/l/h, compared to 5 and 10% inoculums, which yielded 89% each with productivities of 2.0 and 4.8 g/l/h, respectively. A different phenomenon occurred when a 20% inoculum with a 2-day-old inoculum was used, resulting in a higher lactic acid yield of 97%, but with reduced productivity at 3.3 g/l/h (Martínková et al., 1991).

Research by Taleghani et al. (2016) reported that fermenting whey with *L. bulgaricus* for 72 h using different inoculum sizes produced varying lactic acid yields. In their study, using culture sizes of 1–15% resulted in yields of 43.1, 66.3, and 72.7% for culture sizes of 1, 5, and 10%, respectively. However, there was a decrease in lactic acid yield when the culture size was increased to 15% (yield 62.5%). This decrease is attributed to the depletion of substrates (nutrients or sources of energy) in the fermentation broth, leaving insufficient carbon sources for the microorganisms. Similarly, the production of lactic acid from whey with *L. casei* using culture sizes of 1 to 5%, as reported by Panesar et al. (2010), revealed that substrate consumption and lactic acid production increased with an increase in inoculum size up to 2% (v/v). However, there was no significant change in either parameter when the inoculum size exceeded 2%. The maximum lactic acid production, at 33.72 g/l, was achieved using a 2–4% inoculum. The lowest lactic acid production occurred with a 1% inoculum, likely due to the low starter culture density.

### Substrate concentration

Substrate inhibition is a common issue that can decrease the yield and productivity of LA production. It occurs when there is excessive carbon intake during fermentation (Dumbrepatil et al., 2008). High concentrations of substrates can lead to longer lag phases, osmotic stress, cellular lysis, and a decrease in microorganism activity, all of which contribute to a decrease in the effectiveness of LA production (González-Leos et al., 2019).

Researchers have noted that higher substrate concentrations can result in decreased concentration, yield, and productivity of LA produced due to the formation of substrate inhibitors during the fermentation process (Abdel-Rahman, S.E.-D. Hassan,et al., 2019). In their study on lactic acid production using glucose as a substrate in the range of 20–150 g/l, with pH control at 9, they found that at glucose concentrations of 20–100 g/l, the conversion was complete within 9–90 h, resulting in lactic acid concentrations ranging from 19.6 to 96.0 g/l and yields between 0.93 and 0.96 g/g, respectively. The maximum lactic acid concentration was obtained when an initial glucose concentration of 150 g/l was used, resulting in 109.9 g/l of lactic acid after 108 h of fermentation. However, lactic acid productivity decreased with increasing glucose concentrations used (Abdel-Rahman et al., 2019). Therefore, selecting fermentation methods with high substrate concentrations and high LA production is necessary. The use of fed-batch mode is one solution to this problem, as this method can reduce substrate inhibition and produce LA with maximum concentration (Zhang et al., 2011).

In a study by Gómez-Gómez et al. (2015),*Thermo anaerobacter* sp. USBA-018 produced 18.2 mM of LA using a glucose substrate at a concentration of 20 mM. However, the LA concentration decreased as the glucose concentration increased from 40 to 200 mM, with lactic acid concentrations ranging from 15.7 to 13.9 mM (Gómez-Gómez et al., 2015). Similarly, LA production from *L. delbrueckii* mutant Uc-3 using molasses in the concentration range of 110–500 g/l (equivalent to 50–240 g/l of total sugar) showed varying results. When molasses concentrations were in the range of 100–190 g/l, the lactic acid concentration and yield increased to 84.6–166.0 g/l and 0.94–0.95 g/g, respectively. However, when molasses concentrations exceeded 240 g/l, both lactic acid concentration and yield decreased (Dumbrepatil et al., 2008).

### Mixture of sugars as carbon source

LAB are commonly used in lactic acid production due to their efficiency (Abdel-Rahman and Sonomoto, 2016). In LA fermentation, a mixture of sugars is used as a substrate. The LAB strain used typically prefers glucose over other sugars. This preference is because glucose is a simple single sugar that can be easily utilized by microbes, thereby shortening the fermentation time and enhancing the titer, yield, and productivity of the lactic acid. This phenomenon is known as carbon catabolite repression (CCR) (Zaldivar et al., 2001; Tan et al., 2017). Various approacheshavebeen taken to reduce the effect of CCR and increase the effectiveness of LA productivity. These include the utilization of cocultures, genetic engineering, variations in sugar concentration, and the use of inhibitor-resistant strains in LA fermentation (Kim et al., 2009; Cui et al., 2011; Abdel-Rahman et al., 2021).

### Accumulation of by-products

The utilization of hetero-fermentative LAB in the production of LA can reduce the yield of LA due to the presence of by-products such as ethanol and acetic acid in the product (Fig. 3). In addition, the presence of byproducts in LA production requires the separation and purification of the desired LA (Gao et al., 2012). Therefore, using strains that only produce LA is preferable to reduce production costs and increase the efficiency of LA production.

### Final product concentration

The accumulation of LA as the final product in fermentation can lead to a decrease in the pH of the fermentation medium, which can inhibit bacterial growth and reduce the productivity of LA production (Wee et al., 2006). Developing fermentation strategies for LA production under toxic conditions is necessary to overcome product inhibition. Productivity can be improved by periodically removing accumulated LA from the medium and using high cell density (Schiraldi et al., 2003; Zhou et al., 2019). Fed-batch fermentation and pH control have been reported to overcome product inhibition in LA production, but there are still shortcomings such as the presence of acid anions and high osmotic pressure (Cui et al., 2016).

## Purification of LA

LA obtained from fermentation must undergo a separation and purification process to remove contaminants (Bishai et al., 2015). The purification process is complex and expensive, necessitating the development of cost-effective methods to reduce the cost of LA production. Several techniques are used for separating and purifying LA from fermentation media, including precipitation, liquid–liquid extraction, membrane separation, and distillation (Kumar et al., 2019; Din et al., 2021; Li et al., 2021).

Precipitation is commonly used to separate LA from fermentation (Wasewar et al., 2004). media, where LA is recovered from precipitated calcium lactate by adding sulfuric acid. However, this technique is costly and generates solid waste that can pollute the environment, resulting in LA produced at 22 to 44% purity.To obtain highgrade LA, additional treatments such as esterification with alcohol, recovery of the formed ester by distillation, hydrolysis with water, and evaporation are needed (Shreve, 1977; Eyal and Bressler, 1993).

The purification of LA from its medium using solvent extraction/liquid–liquid extraction technique is a separation process that involves the reaction between the extractant and the extracted material. Solvent extraction is used to separate carboxylic acids in the solution and the separation of LA. LA is extracted from the fermentation broth by the extractant used, and pure LA is obtained by reverse extraction of extract liquor. The efficiency of LA separation with conventional organic solvents is very low (10^-5^ to 10 mol/l based on the concentration of unreacted LA at equilibrium), so developing a complex solvent-extracting method or other techniques is needed (Han and Hong 1996; Han et al. 2000).

Membrane technology is widely used for purifying LA from fermentation broth due to its ease of use and high separation efficiency. Various membrane technologies, such as ultrafiltration and nanofiltration, have been reported to obtain high-purity LA (> 99.5%). However, this technique can be costly (Lee et al., 2017).

Distillation is another method used for purifying LA, yielding high-purity LA. However, distillation may require high energy due to the high boiling point of lactic acid (122˚C) and may result in the formation of dimers. Reactive distillation, which combines distillation with esterification and hydrolysis, can produce high-purity LA yields (Kumar et al., 2006; Komesu et al., 2015). Combining reactive distillation with other methods, such as hybrid short path distillation coupled with reactive distillation, can achieve better separation and high-purity LA (Andrea et al., 2016).

## Conclusion

LA production through microbial fermentation represents a sustainable and ecofriendly alternative to chemical synthesis, offering potential benefits to various industries. The use of renewable and low-cost substrates, as well as mixed microbial cultures, has improved efficiency and cost-effectiveness in LA production. However, challenges such as inhibition, by-product accumulation, and high costs remain.

Innovative approaches, including membrane technology, can help improve the efficiency of LA production. Additionally, the development of more efficient microorganisms can increase yield and productivity while reducing contamination risks. Overall, microbial fermentation holds promise for sustainable LA production, with the potential to significantly reduce environmental impact and provide a cost-effective source for various industries.
